# Label-Free Plasmon-Waveguide Resonance Analysis of Mucin Network Remodeling by Cationic Polymers

**DOI:** 10.1021/acsmeasuresciau.6c00012

**Published:** 2026-04-09

**Authors:** Shilpa E. George, Amanda M. Ratajczak, Uday B. Kompella

**Affiliations:** † Department of Pharmaceutical Sciences, 129263 University of Colorado Anschutz Medical Campus, Aurora, Colorado 80045, United States; ‡ 683164 Mainline Scientific LLC, Malvern, Pennsylvania 19355, United States; § Department of Ophthalmology, University of Colorado Anschutz Medical Campus, Aurora, Colorado 80045, United States; ∥ Department of Bioengineering, University of Colorado Anschutz Medical Campus, Aurora, Colorado 80045, United States; ⊥ Colorado Center for Nanomedicine and Nanosafety, University of Colorado Anschutz Medical Campus, Aurora, Colorado 80045, United States

**Keywords:** plasmon-waveguide resonance spectroscopy, PWR spectroscopy, mucin, Cationic polymers, chitosan, polyethylenimine, poly-l-lysine

## Abstract

Surface plasmon resonance (SPR) quantifies biomolecular interactions using *p-*polarization. Plasmon-waveguide resonance (PWR) adds *s-*polarization, yielding concurrent readouts of refractive*-*index changes perpendicular and parallel to interfacial films and, thus, optical anisotropy. We validated label*-*free PWR for mucin and quantified how cationic polymers remodel immobilized anionic mucin via a graphical mass/structure decomposition. Reproducible system performance was confirmed with air, water, and PBS; mucin showed concentration*-*dependent angle shifts with the expected order air < water < PBS < mucin in both polarizations. A sensitivity factor (*Sf* = Δ*s*/Δ*p*) of 0.33 from buffer transitions enabled the resolution of mass versus structural contributions. Mucin (0.1% w/v) was probed with chitosan, branched polyethylenimine (PEI), and poly-l *-*lysine (PLL) (each 0.1% w/v), with baseline mucin responses being mass dominated (∼77%) with a smaller structural component (∼23%). PEI produced large positive shifts (Δ*p* = 310.3 ± 56.8; Δ*s* = 300.2 ± 54.8 millidegrees), yielding a mass*-*dominated response (∼65%) with a substantial structural term (∼35%) oriented toward increased anisotropy relative to mucin, consistent with strong polymer accumulation on the mucin film. Chitosan caused a moderate increase in Δ*p* (82.3 ± 40.4) with negligible Δ*s* (11.2 ± 7.4 millidegrees), producing a smaller mass contribution relative to PEI and a notable structural contribution (∼38%); the vector orientation indicates decreased anisotropy with moderate mass addition. In contrast, PLL induced negative shifts (Δ*p* = −101.6 ± 15.6; Δ*s* = −31.1 ± 23.8 millidegrees), reflecting net mass loss with a minor structural component (∼14%), consistent with partial mucin desorption/thinning. Dual*-*polarization PWR thus resolves polymer*-*specific outcomes, including decreased anisotropy with mucoadhesive restructuring (chitosan), mass*-*dominant accumulation with increased anisotropy (PEI), and barrier weakening via partial desorption (PLL). Mucin*-*coated PWR provides a reproducible, label*-*free platform to deconvolve mass versus structural effects in mucin-polymer interactions and to rapidly screen mucoadhesive and penetration*-*enhancing excipients for mucosal drug delivery.

## Graphical abstract

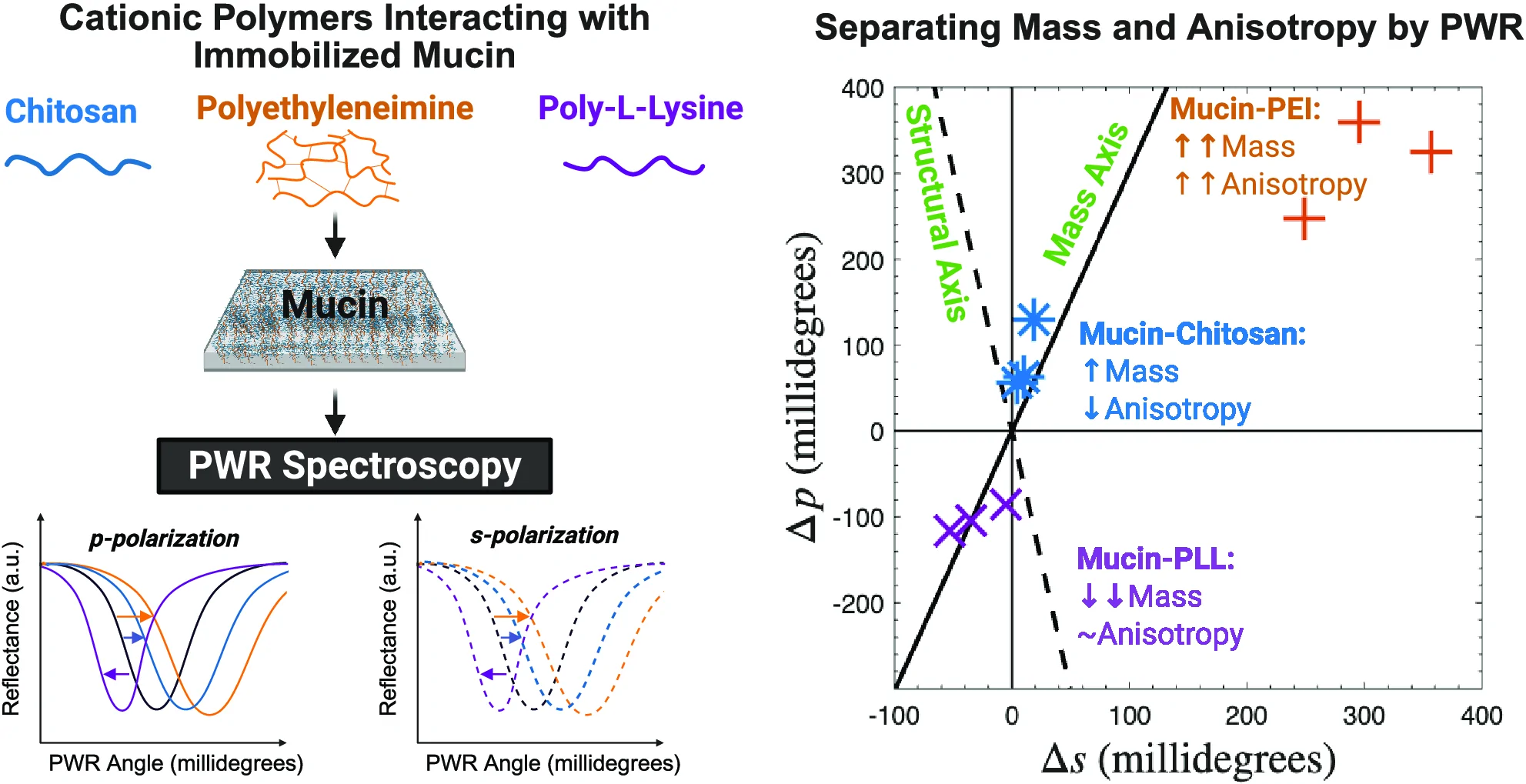

## 1 Introduction

Mucoadhesive polymers are often incorporated in drug delivery systems to increase drug residence time and delivery across mucosal barriers.^1–3^ These polymers interact with mucin, an anionic biopolymer, which is a major constituent of the mucosal layer, and understanding these interactions at a molecular level is critical in the development of an effective mucoadhesive drug delivery system.^4^

Over the years, several techniques, including surface plasmon resonance (SPR) spectroscopy, have been developed to probe molecular interactions in real time. SPR quantifies these interactions by detecting variations in the refractive index caused by the binding of analytes with immobilized ligands.^5^ It has also been widely used to obtain quantitative insights into the mucoadhesive behavior of polymers. For example, Bravo-Osuna et al. characterized the interfacial interaction of transmembrane ocular mucins with various polymers using SPR. They found that among all the polymers tested, only the positively charged chitosan showed measurable interaction with mucin, confirming its known mucoadhesive nature. Whereas the negatively charged carboxymethyl cellulose (CMC) and hyaluronic acid (HA), and the neutral hydroxypropyl methylcellulose (HPMC), showed no detectable mucoadhesion under the same conditions.^6^ Takeuchi et al. used SPR to demonstrate the strong mucoadhesive behavior of positively charged chitosan (MW 20,000 and 150,000) and negatively charged Carbopol (971 PNF and 974 PNF) with porcine mucin.^7^ Despite this progress, traditional SPR techniques often come with certain limitations. First, the preparation of a reliable, mucin-coated SPR sensor chip can be laborious and time-consuming. For instance, Bravo-Osuna et al. incubated mucin onto gold chips overnight, followed by a 12-h water wash to stabilize the film, taking an overall 24 h of preparation before any binding assay could be performed.^6^ Second, SPR captures data using a single polarization mode (*p*-polarization), which probes refractive index changes perpendicular to the sensor/sample surface.^8^ This limits the ability of SPR to directly identify conformational changes in the bound molecules. Although several studies have shown conformational changes indirectly from SPR signal patterns or kinetic shifts, these interpretations are based on secondary effects such as altered mass density, hydration, etc., rather than direct assessment of structural changes.^9–11^

Plasmon-waveguide resonance (PWR) spectroscopy addresses the limitations of SPR by enabling the detection of biomolecular interactions using dual polarization modes (both *p*- and *s*-polarizations) while eliminating the need for lengthy immobilization steps. Although ligand immobilization times can vary depending on the experimental setup, immobilization generally takes about 30 min in a typical PWR experiment.^12,13^ Another advantage of PWR is the addition of the *s-*polarization mode that enables probing parallel to the sensor surface, which is more sensitive to surface properties. The combination of *s*- and *p*-polarizations can provide information about the anisotropy of molecules at the sensor surface.^14,15^ Broadly speaking, there are two primary variants of PWR: in one, the prism is rotated and a spectrograph of the intensity of the detected light versus incident angle is returned; in the other, the prism orientation is fixed while the wavelength of the incident laser is varied.^16–18^ In the latter configuration, the returned spectrograph shows the intensity of reflectance versus wavelength of the laser source. This study focused on the first method, which involves rotating the prism relative to a single laser source of a fixed wavelength (Figure 1a). Any change in the refractive index of the sample (stemming from changes that arise because of molecular interactions on the surface of the sensor chip) causes the resonance angle to shift, with positive and negative shifts indicating an increase and a decrease in refractive index, respectively. Plotting this shift in resonance angle (PWR angle) over time provides a view of how the sample is changing over time and allows for monitoring molecular interactions in real time.^19–22^ A comparison of *p-* and *s-* polarization shifts will further inform anisotropy and potential differences in molecular orientation in a thin sample on the PWR prism.^13,14,17,20^

**Figure 1:**
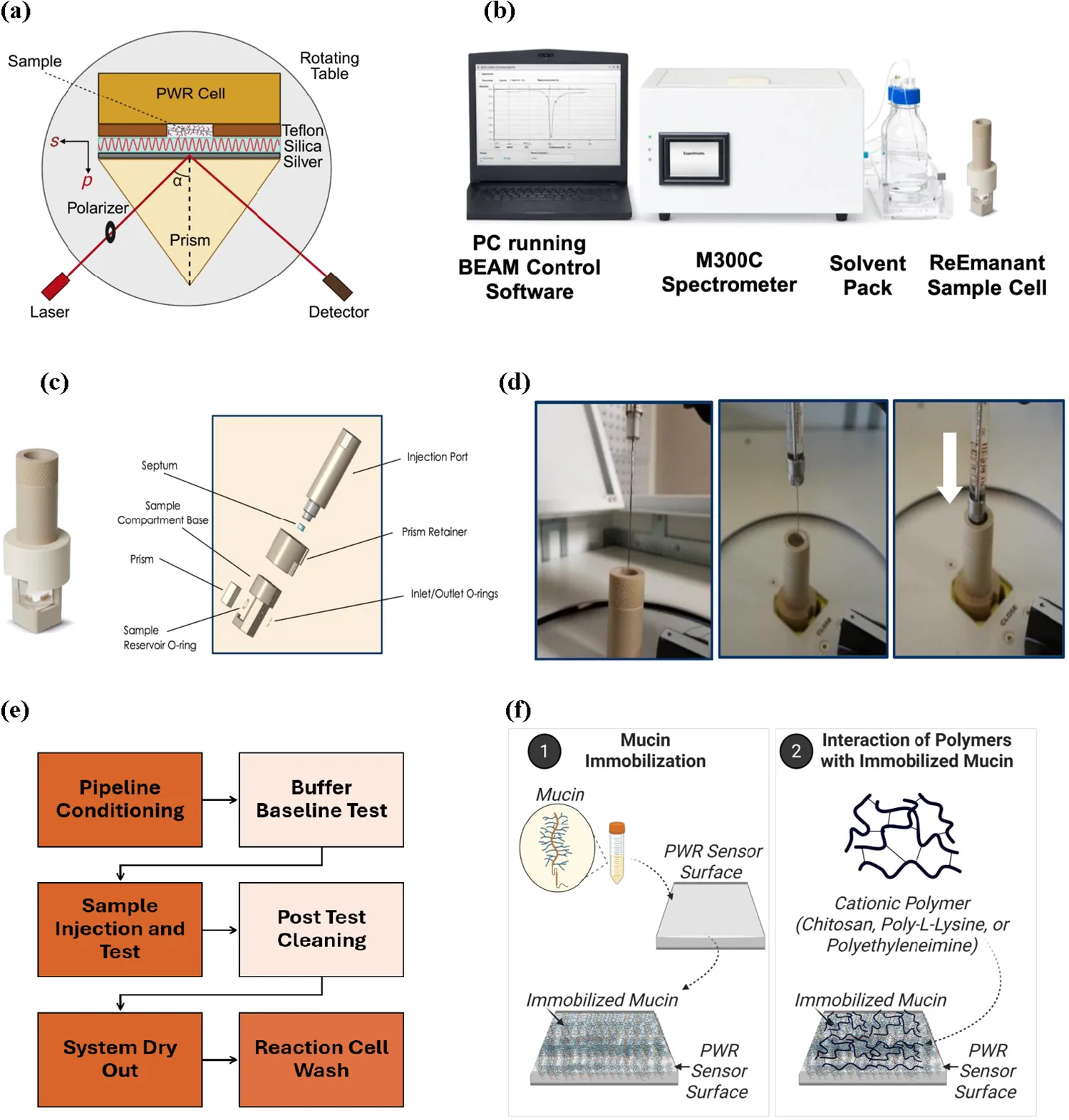
(a) Schematic representation of the working principle of PWR spectroscopy. The angle of incidence (α) is adjusted by rotating the prism relative to a fixed-wavelength laser source (632.8 nm red light from a helium–neon laser), and the polarized beam is directed onto the sensor surface. The reflected light is detected to capture resonance conditions. Molecular interactions at the sensor surface alter the local refractive index, which causes a shift in the resonance angle. By tracking these angle shifts over time, binding events can be monitored in real time. Panel (a) is adapted from ref 15, Rascol et al., Molecules, licensed under CC BY 4.0. (Created in BioRender. George, S. (2026) https://BioRender.com/nion9ae) (b) Diagram of the Emanant PWR Spectroscopy Instrument. (c) Breakdown of a ReEmanant Sample Cell (d) To inject a sample, a syringe is inserted into the Injection Port (left), lowered until the needle tip contacts the Septum (middle), and then pushed downward to pierce the Septum with the needle (right). (e) The standard workflow typically includes a series of steps for a test run. Steps highlighted against a light background are optional. (f) Schematic representation of the methodology used in this study to immobilize mucin on the PWR sensor surface. Mucin solution was introduced onto the PWR sensor surface, and scans were taken until immobilization or no significant change in the resonance angle (PWR angle) was observed. This was followed by the introduction of chitosan, poly-l-lysine (PLL), or polyethylenimine (PEI) solution. (Created in BioRender. George, S. (2026) https://BioRender.com/wxosinb).

In this study, we validated a PWR spectroscope for the binding of reference materials as well as anionic porcine gastric mucin and evaluated the interactions of mucin cationic polymers commonly used in drug delivery applications. Specifically, chitosan, poly-l-lysine (PLL), and polyethylenimine (PEI) were selected as the cationic polymers based on their distinct structures and functional chemistry.^23–27^ Chitosan is a natural linear polysaccharide, PLL is a linear polyamino acid, and the PEI used is a synthetic branched polymer. Together, these polymers offer a range of structural rigidity, branching, and functionality, which would help in understanding the influence of molecular architecture in mucin binding and structural reorganization. Although earlier studies have examined mucin-polymer interactions using techniques such as SPR, this present work takes a new approach by utilizing the dual polarization modes of PWR spectroscopy to characterize not just the interaction of the polymers with mucin but also the anisotropy of these interactions in real time. To the best of our knowledge, this is the first application of PWR spectroscopy to characterize mucin-polymer interactions, informing how cationic polymers interact with and reorganize mucin at the interface.

## 2 Experimental Section

### 2.1 Materials and Buffers

Na_2_HPO_4_·7H_2_O, KH_2_PO_4_, NaCl, and KCl were purchased from Thermo Fisher Inc. (Waltham, MA). Partially purified mucin from porcine stomach, Type III (bound sialic acid 0.5–1.5%) (M1778, CAS Number: 84082–64–4), chitosan (poly­(d-glucosamine) (medium molecular weight, 190,000–310,000 Da) (Cat. No. 44,8877, CAS 9012–76–4)), polyethylenimine (*M*_n_ ∼ 60,000, M_W_ 750,000) (Cat. No. P3143, CAS 9002–98–6), and poly-l-lysine (MW: 150,000–300,000 Da) (Cat. No. P8920, CAS 25988–63–0) were all obtained from Sigma-Aldrich, St. Louis, MO, USA. All the above materials, except mucin, were stored at room temperature (RT). Mucin was received as a lyophilized powder and was stored at 2–8 °C until use.

A 10 times concentration of phosphate buffer saline (PBS) solution was prepared using Na_2_HPO_4_·7H_2_O (14.30 g), KH_2_PO_4_ (2.45 g), NaCl (80.07 g), KCl (2.01 g), and double-distilled water (ddH_2_O). The stock solution was then diluted to a 1× concentration with ddH_2_O, and pH was adjusted to 7.4, using HCl and measured using a pH meter (Mettler-Toledo International Inc., Columbus, OH, USA). The final solution was stored at 2–8 °C until use. A 0.1% solution of acetic acid was prepared by mixing glacial acetic acid (≥99.7%, ACS grade) with ddH_2_O and stored at RT until use.

### 2.2 Preparation of Mucin

Varying concentrations of porcine gastric mucin (hereafter termed mucin) solutions ranging from 0.05% to 2% w/v were used across different experiments. These concentrations were selected to reflect physiologically relevant conditions of mucin. Mucin solutions (0.05, 0.1, 0.2, 0.5, 1, and 2% w/v) were prepared in 1× PBS (pH 7.4) and incubated overnight at ∼4 °C for adequate swelling and solubilization. These solutions were equilibrated at RT (around 23 to 25 °C) for 30 min prior to the experiment. A fresh batch of mucin was prepared for each experiment, and the shelf life of the prepared mucin solution was fixed at 24 h, based on the results of the pH stability study (Supporting Information Table S.1).

### 2.3 Preparation of Polymer Solutions

The polymeric test solutions consisted of chitosan (**linear polymer**), polyethylenimine (PEI) (**branched polymer**), and poly-l-lysine (PLL) (**linear polymer)**. All polymers were used at 0.1% w/v to directly compare their interaction behaviors at a standard formulation-relevant concentration commonly used in mucosal drug delivery systems. Chitosan, supplied as a white powder, was dissolved in 0.1% (v/v) acetic acid and incubated overnight at RT with continuous stirring to solubilize the polymer. PEI was supplied as a highly viscous aqueous solution (50% (w/v)) and was diluted to 0.1% (w/v) in PBS. PLL was supplied in an aqueous base by the manufacturer and was used as received without further dilution or modifications. All solutions were stored at RT and used within 1 week of preparation.

### 2.4 Physicochemical Characterization of Reference and Test Solutions

The pH of the solvents (PBS, 0.1% v/v acetic acid) and test solutions (0.1% w/v mucin, 0.1% w/v chitosan, 0.1% v/v PEI, and 0.1% v/v PLL) were measured at room temperature using a calibrated pH meter.

ζ-Potential, hydrodynamic diameter, and polydispersity index (PDI) were assessed for both reference and analyte solutions using dynamic and electrophoretic light scattering (Zetasizer Nano ZS; Malvern Panalytical, Malvern, UK).

### 2.5 Emanant Spectrometer System

The Emanant PWR Spectroscopy System (Model M300C, input DC at 24 V,1.5A) (Mainline Scientific LLC, Malvern, PA, USA) was used for the PWR spectroscopy measurements. This system is based on the fixed incident wavelength/rotating prism approach to PWR with a 632.8 nm helium–neon red laser as the excitation source. The compact benchtop design enables to collect molecular interaction data in real time and in-house with high-resolution using label-free samples (Figure 1b). The resonance is detected using a high-refractive-index glass prism sensor chip with a hydrophilic surface, which supports plasmon-waveguide coupling and allows direct adsorption of biomolecules without prior surface modification or sample labeling. This, in turn, reduces the time and costs associated with running an assay. The ReEmanant sample cell, a small module that is inserted into a port at the top of the Emanant system before each run, houses the sensor chip and the sample reservoir (Figure 1c). The sample cell accommodates low-volume injections, requiring as low as 10 μL per sample injection. Buffer solutions are pumped into the system using the instrument’s internal pump, while the samples are manually injected into the sample reservoir, reducing potential contamination of the fluidic path upstream of the sample loop (Figure 1d).

BEAM software (Version 3.0.0.26, Mainline Scientific LLC, Malvern, PA, USA) was used for instrument control and data acquisition. The software allows one to choose between real-time control or preprogrammed protocols for executing experimental workflows and visualizes experimental data both in real time during an experiment and after an experiment’s conclusion. The PWR sensor chips used in this system are reusable, provided that the chip-cleaning protocol is performed between tests.

### 2.6 PWR Spectroscopy

#### 2.6.1 Reference Scans

Instantaneous reference scans of air, water, and PBS were obtained at the start of each experiment. These measurements were used for normalization purposes to account for slight variations in initial prism positioning, which ensured reproducibility across experiments and reduced the run-to-run variability. Air scans were performed in ’sample injection’ mode, while water and PBS scans were conducted in ’baseline buffer scan’ mode. 1× PBS (pH 7.4) was used as the second baseline since mucin test solutions were prepared in PBS. Pipeline conditioning was performed with distilled water before each experiment to fill the pipelines of the instrument at a speed of 5 mL/min and a volume of 5700 μL. Baseline measurements with water and PBS were performed by pumping each solution through the PWR pipeline at a flow rate of 5 mL/min. A volume of only 2850 μL was used for water injection since the pipeline was already filled with water during the pipeline conditioning step. In contrast, 5700 μL of PBS was used to ensure complete flushing of water from the pipeline and to prevent dilution of the PBS sample during measurement. The standard experimental workflow is outlined in Figure 1e.

#### 2.6.2 Mucin Immobilization

Mucin (100 μL), ranging from 0.05% to 0.5% w/v, was injected directly into the reaction cell using a 26 G needle of 2 in. length connected to a Hamilton syringe and allowed to immobilize for 30 min. During this period, scans were continuously obtained between the *p-* and *s-*polarization modes, at predetermined intervals (Figure 1f).

#### 2.6.3 Polymer Injection

Once mucin was equilibrated on the sensor surface, 100 μL of the polymer solutions (chitosan, PLL or PEI) were injected into the reaction cell in a manner similar to mucin injection. The *p-* and *s-*polarization scans were performed for 30 min or until no significant change in the PWR angle was observed. The reaction cell remained undisturbed throughout the experiments (Figure 1f).

### 2.7 Data Analysis

The resonance position shifts (RPS) for mucin and the polymer solutions were normalized to the baseline established by using the reference sample (PBS), recorded prior to mucin injection. Polymer-induced resonance shifts from immobilized mucin were calculated relative to the final mucin reading recorded immediately before polymer addition.

Data are presented as the average of three experiments with error bars representing the standard deviation (presented as mean ± SD). One-way ANOVA was used to determine the significance of the peak shift from the reference sample (PBS) or immobilized mucin. For post hoc analysis following ANOVA, Tukey’s test was applied. Statistical significance was determined at a 95% confidence interval. All the statistical analyses were conducted using GraphPad Prism software (Version 10.3.1).

## 3 Results and Discussion

### 3.1 Stability of Mucin Preparations in Terms of pH

The pH of mucin solutions at varying concentrations (0.05–2%) was monitored over time (about 8 days or 196 h) to assess the stability of mucin with storage at RT and refrigerated (∼4 °C) conditions. PBS served as a control.

At RT, initially, all mucin test solutions had a pH between 7.34 and 7.41, which was in good agreement with that of the control (pH 7.4). However, over the days of the study, a concentration- and time-dependent decline in the pH of the mucin solutions was observed relative to the control solution. The control and mucin solutions with low concentrations (0.05% and 0.1% w/v) exhibited minimal decrease in pH (pH range: 7.17 to 7.41, SD ≤ 0.08), while the 1% and 2% mucin solutions showed significant decrease in pH (pH range: 6.81 to 7.37, SD > 0.206) over 8 days at RT, with the drop in pH below 7, observed after 72 h of mucin preparation.

Mucin samples stored under refrigerated conditions (CV% = 1.06 to 1.28) showed better pH stability than the samples stored at RT (CV% = 1.12 to 3.07). All formulations at refrigerated conditions, including 1% and 2% mucin, maintained a pH of 7.2 ± 0.03 throughout the 8-day period, with standard deviations of ≤0.09. It is worth noting that the occasional dip in pH at intermediate time points (e.g., 144–168 h), possibly due to temperature differences or exposure to air during pH measurement, was followed by recovery, suggesting better buffering or slower degradation at reduced temperatures.

Due to the high susceptibility of mucin solutions to pH changes at RT, mucin solutions used in this study were stored under refrigerated conditions (∼4 °C). Due to the time-dependent decrease in pH observed in mucin samples under both RT and refrigerated storage conditions, all mucin solutions used in this study were freshly prepared and not utilized beyond 24 h from the time of preparation *(refer* Supporting Information Table S.1).

As previously stated, the 0.05% and 0.1% (w/v) mucin solutions exhibited the least variability in pH under both RT (CV% = 1.12 for each) and refrigerated storage (CV% = 1.06 and 1.15 for 0.05% and 0.1% w/v, respectively). The 0.1% w/v mucin solution was selected for polymer interaction studies, as its higher concentration produced a more pronounced RPS relative to the 0.05% mucin and PBS control (Figure 3c).

### 3.2 Unique Optical Properties of Reference Materials and Mucin

PWR spectroscopy conducted on the reference samples (air, water, and PBS) and the test mucin sample (0.1% w/v) revealed distinct ranges in PWR angles for each sample, with millidegree precision. The angles increased in the order of air < water < PBS < 0.1% mucin for both *p*- and *s*-polarization modes (Figure 2a). The RPS of PBS from water was higher in *p*-polarization due to the increased refractive index of PBS, whereas the RPS in *s*-polarization between the same reference materials was much lower. However, in the case of mucin, the RPS values in both *p*- and *s*-polarization modes were much larger than those of the reference scans (Figure 2b).

**Figure 2:**
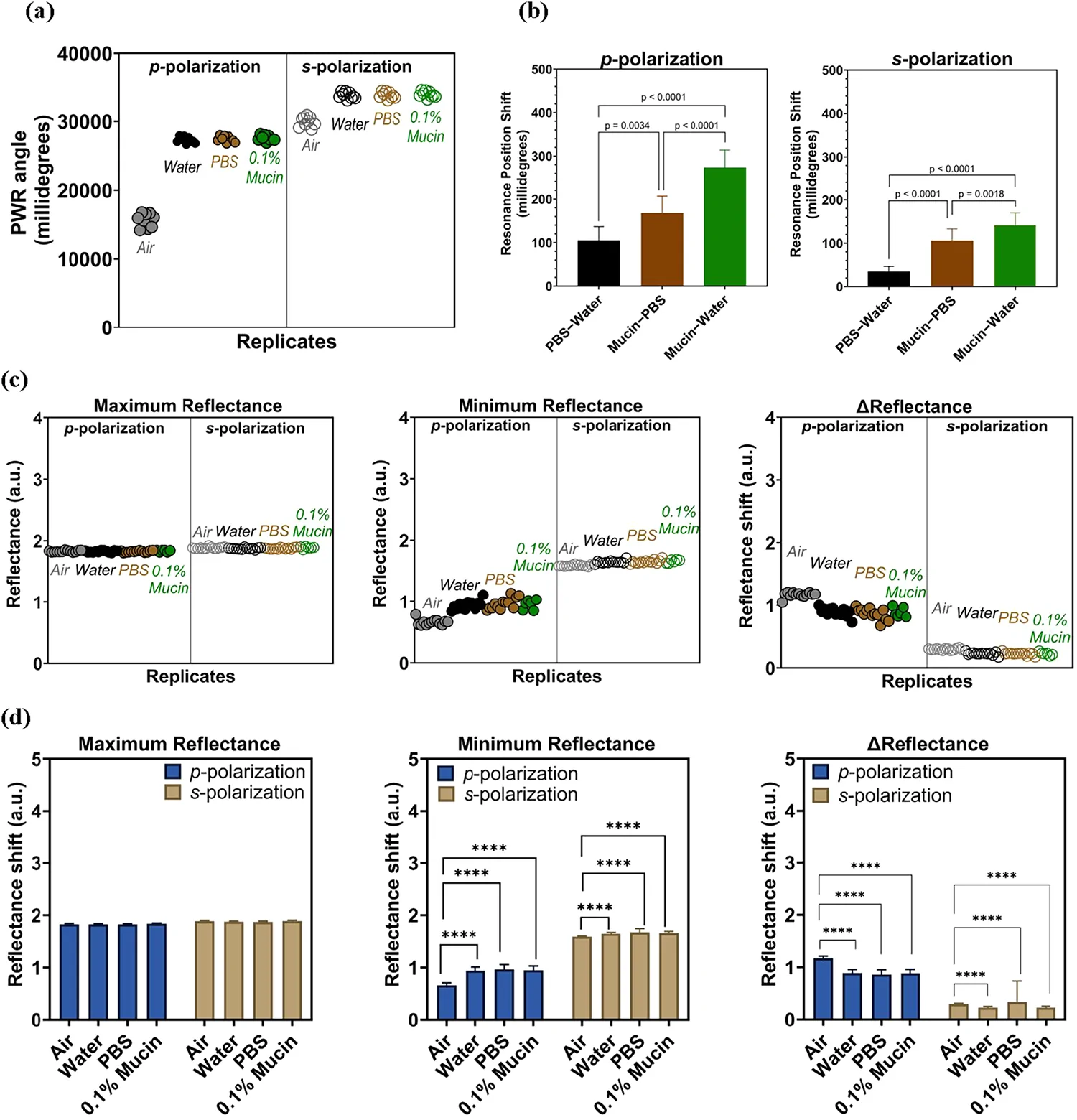
Interaction of reference materials (air, water, and PBS) and 0.1% mucin with the PWR sensor. (a) PWR angles obtained for reference materials and 0.1% w/v mucin across multiple experiments. A progressive increase in PWR angle was observed from air to mucin, following the order: air < water < PBS < 0.1% mucin, in both p- and s-polarization modes. More pronounced changes were observed under p-polarization. (b) Resonance position shift (RPS) measurements of PBS from water (PBS-Water), 0.1% mucin from PBS (Mucin-PBS), and 0.1% mucin from water (Mucin-Water) measured under p- and s-polarization modes corroborate the results shown in panel (a). Significant increase in resonance position (calculated from the PWR angle) was seen for PBS compared to water, and for mucin compared to both PBS and water, indicating that each test sample had distinct PWR angles (water < PBS < 0.1% mucin). This effect was more prominent under p-polarization than s-polarization. Statistical significance of RPS differences between groups was determined using one-way ANOVA with Tukey’s post hoc test (95% confidence interval). The error bars denote standard deviation, with *n* = 9. Statistical significance is represented as follows: *p* < 0.05 (*), *p* < 0.01 (**), *p* < 0.001­(***), *p* < 0.0001­(****); ns, nonsignificant. (c, d) Maximum reflectance (peak height), minimum reflectance (resonance dip depth), and reflectance shift (difference between maximum and minimum reflectance) for air, water, PBS, and mucin. Maximum reflectance remained relatively constant across all conditions. Minimum reflectance increased from air to mucin, and the reflectance shift indicated a decreasing dip depth. Statistical analysis showed significant changes in minimum reflectance relative to air (baseline) for all the samples tested, but no significant differences from water to PBS or mucin and PBS to mucin (*p* > 0.7; not shown in the plots) were observed.

In addition to the PWR angle changes, variations in the reflectance values (maximum, minimum, and the difference between them (reflectance shift)) were also recorded (Figure 2c,2d). A trend, consistent with the PWR angle shifts, was observed in the minimum reflectance (i.e., the depth of the resonance dip), which increased in the order of air, water, PBS, and mucin. This indicates a progressive decrease in peak depth as we move from reference samples to mucin, suggesting an indirect measurement of increasing refractive index. On the other hand, the maximum reflectance (the highest reflectance value near the onset of the resonance curve) remained relatively consistent across all the study samples, with no significant difference between the samples. The Δ reflectance (the difference between the maximum and minimum peak values) corroborated the trend observed in minimum reflectance, confirming that the peak was deepest with air and became progressively less pronounced from air to mucin. While these values may help illuminate the optical behavior of the reference samples and analytes, further investigation into these trends is required and thus the remainder of this study focuses solely on PWR angle shifts to understand the behavior of mucin-polymer interactions. This is because angle-based interactions are more sensitive when studying molecular interactions at the surface level, compared to reflectance measurements (Figure 2b–2d).

### 3.3 Reproducibility of PWR Spectroscopy in *p*- and *s*- Polarization Modes

For all reference samples and analytes tested, a reproducible and distinct spectrum was obtained. The spectral profile depended on the initial positioning of the reaction cell containing the PWR sensor. While the absolute spectral positions varied slightly each day due to this positioning, the values remained consistent within a characteristic range for each sample. The air peak was observed between 14,200 and 16,800 millidegrees for *p*-polarization and 29,000–31,000 millidegrees for *s*-polarization, serving as the experimental baseline. The largest deviation in PWR angle occurred between air and water, with water peaks ranging from 26,600 to 27,900 millidegrees (*p*-polarization) and 33,150–34,500 millidegrees (*s*-polarization), respectively. Although water and PBS showed similar ranges, PBS consistently exhibited a higher PWR angle than water regardless of the equilibration time. The shift between PBS and water was approximately 105 millidegrees for *p*-polarization and 35 millidegrees for *s*-polarization. The coefficients of variation (CV%) for the reference scans (air, water, and PBS) range from 1.39% to 6.44% for both polarization modes, with *s*-polarization consistently exhibiting lower CV% values than *p*-polarization. Among the reference samples, air exhibited the highest variability, followed by water and then PBS, which showed the lowest CV%. Upon mucin immobilization on the sensor surface, a more pronounced spectral shift was observed, which was around 169 millidegrees for *p*-polarization (CV% = 1.46) and 106 millidegrees (CV% = 1.32) for *s*-polarization.

### 3.4 Dose–Response Relationship for Mucin-PWR Sensor Interactions

The Emanant PWR spectrometer demonstrated sensitivity not only to different analyte materials but also to subtle changes in the concentration of the same sample. We tested mucin concentrations ranging from 0.05% to 0.5% and observed a gradual increase in the PWR angle with an increasing concentration of the test solution (Figure 3a,3b). A positive shift in the resonance position relative to the PBS baseline was observed across all concentrations, with the highest shift recorded for 0.5% mucin. For *p-*polarization, the average shift ranged from 146.0 ± 21.8 to 236.4 ± 33.5 millidegrees (CV% = 7.3 to 14.9, *n* = 3), while for *s*-polarization, it ranged from 81.6 ± 17.4 to 126.4 ± 31.0 millidegrees (CV% = 12.3 to 24.5, *n* = 3) as the mucin concentration increased (Figure 3c). The trend was reproducible and indicative of the capacity of the instrument to detect gradual changes in the PWR angle. The RPS for *p*-polarization was considerably higher than that of *s*-polarization, with a difference of 110 millidegrees for the highest concentration of mucin (0.5% w/v) between *p*- and *s*-polarization modes, consistent with an increase in the adsorbed mass of mucin. In contrast, the difference in *s*-polarization RPS difference between mucin concentrations decreased at higher concentrations, suggesting that following the formation of the mucin layer, organization did not change much. Instead, the higher mucin concentrations primarily contributed to an increase in optical density, with minimal additional structural reorganization when compared to lower mucin concentrations. A limitation of this study is the narrow range of mucin concentrations measured. Expanding the range of concentrations would provide a more comprehensive understanding of the sensitivity of the instrument to minor changes in concentration.

**Figure 3:**
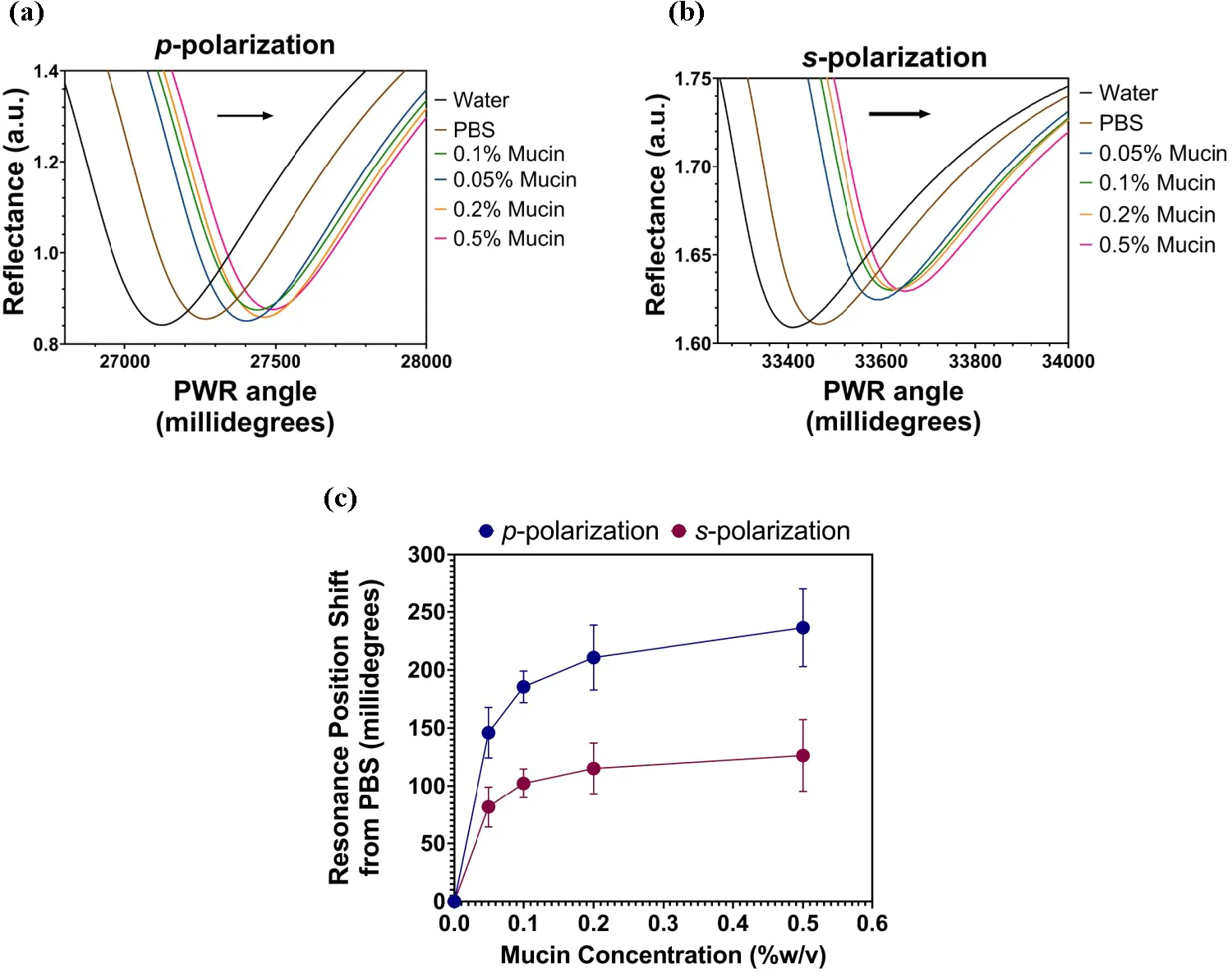
Concentration-dependent interaction of mucin with the PWR sensor surface. (a, b) PWR angle changes observed for 0.05%, 0.1%, 0.2%, and 0.5% w/v mucin, showing a gradual increase in angle with increasing mucin concentration, in *p*- and *s*-polarization modes, respectively. (c) Resonance position shifts of 0.05%, 0.1%, 0.2%, and 0.5% w/v mucin relative to PBS under *p*- and *s*-polarization modes. Both polarizations showed a nonlinear increase in the resonance position shift with increasing mucin concentration. The shifts were more pronounced in p-polarization, showing greater sensitivity to mass accumulation at the surface, while *s*-polarization showed smaller changes corresponding to structural rearrangements within the mucin layer. Among all tested concentrations, 0.1% w/v mucin showed the least variability and was used for subsequent polymer interaction studies. Data are shown as mean ± SD for *n* = 3.

The 0.1% w/v mucin concentration was selected for all subsequent polymer interaction studies as the solution maintained stable pH over the experimental period (Section 3.1) and produced consistent RPS responses with lower variability compared to both lower (0.05%) and higher (≥0.2%) mucin concentrations (Figure 3c). While higher concentrations showed larger mean shifts, they were accompanied by greater variability and pH instability, making 0.1% mucin the most reliable and experimentally robust choice for subsequent measurements. Furthermore, this concentration is within the range used in prior in vitro mucoadhesion studies as a physiologically relevant mimic of hydrated mucin films at epithelial surfaces.^28,29^

### 3.5 Interaction of Cationic Polymers with Immobilized Mucin

Mucin (0.1% w/v) was immobilized on the PWR sensor surface for 30 min prior to introducing the polymers. During this period, the PWR angle was captured in real time, showing an overall gradual and continuous increase in the angle relative to the baseline PBS angle, denoting an ongoing immobilization of mucin onto the PWR sensor surface (Figure 4a). A gradual shift in the PWR angle was observed over an immobilization period of 2 h, without reaching a plateau. Despite the constant rise in the angle, the rate of change in the PWR angle decreased significantly after ∼30 min of immobilization. Based on this observation, a 30 min immobilization period was selected for all subsequent studies, since it was considered sufficient to obtain a representative mucin coating and to maintain experimental consistency.

**Figure 4:**
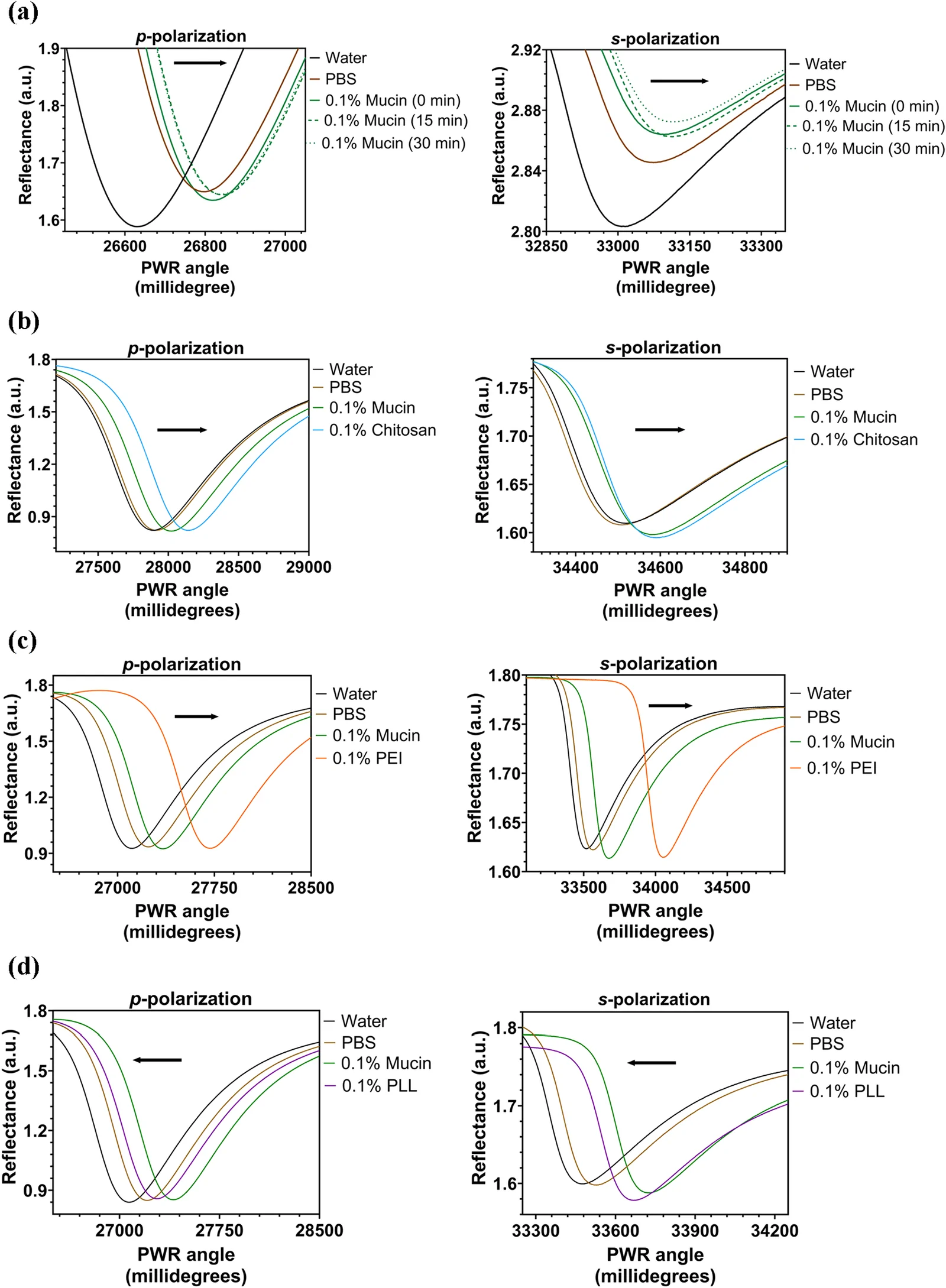
(a) PWR angle shift for 0.1% mucin at 0, 15, and 30 min shows a gradual increase over time, indicating continuous surface immobilization. (b–d) Interaction of 0.1% mucin with test polymers after 30 min of injection: (b) chitosan caused a slight PWR angle increase, more pronounced in p-polarization; (c) PEI induced a strong PWR angle increase in both polarizations; (d) PLL caused a significant decrease in PWR angle compared to mucin.

Chitosan, polyethylenimine (PEI), and poly-l-lysine (PLL) (0.1% w/v) were introduced onto immobilized mucin, and their interactions were monitored for 30 min. The selected polymer concentration falls within the standard range commonly used in the formulations. The same concentration was applied to all polymers to enable a direct comparison and standardization. This concentration was sufficient to generate quantifiable interaction signals. An interaction period of 30 min was chosen based on time-course experiments, which showed that most mucin-polymer binding and structural changes occurred within this window, after which the signal reached a near-steady state. This duration was therefore sufficient to capture the mucin-polymer interaction behavior.

Introduction of these cationic polymers onto immobilized mucin resulted in a rapid shift in the PWR angle compared to the mucin baseline (defined as the value of immobilized mucin at the 30 min time point) (Figure 4b–4d). Chitosan introduction led to a moderate positive shift in *p*-polarization with little or no net shift in *s*-polarization (Figures 4b and 5a,5d). PEI, in contrast, produced large positive shifts in both *p*- and *s*-polarization modes (Figures 4c and 5b,5e). PLL behaved differently from both chitosan and PEI, producing negative shifts in the PWR spectrum, with peak positions falling below that of the mucin baseline, particularly in the *p*-polarization mode (Figures 4d and 5c,5f).

**Figure 5:**
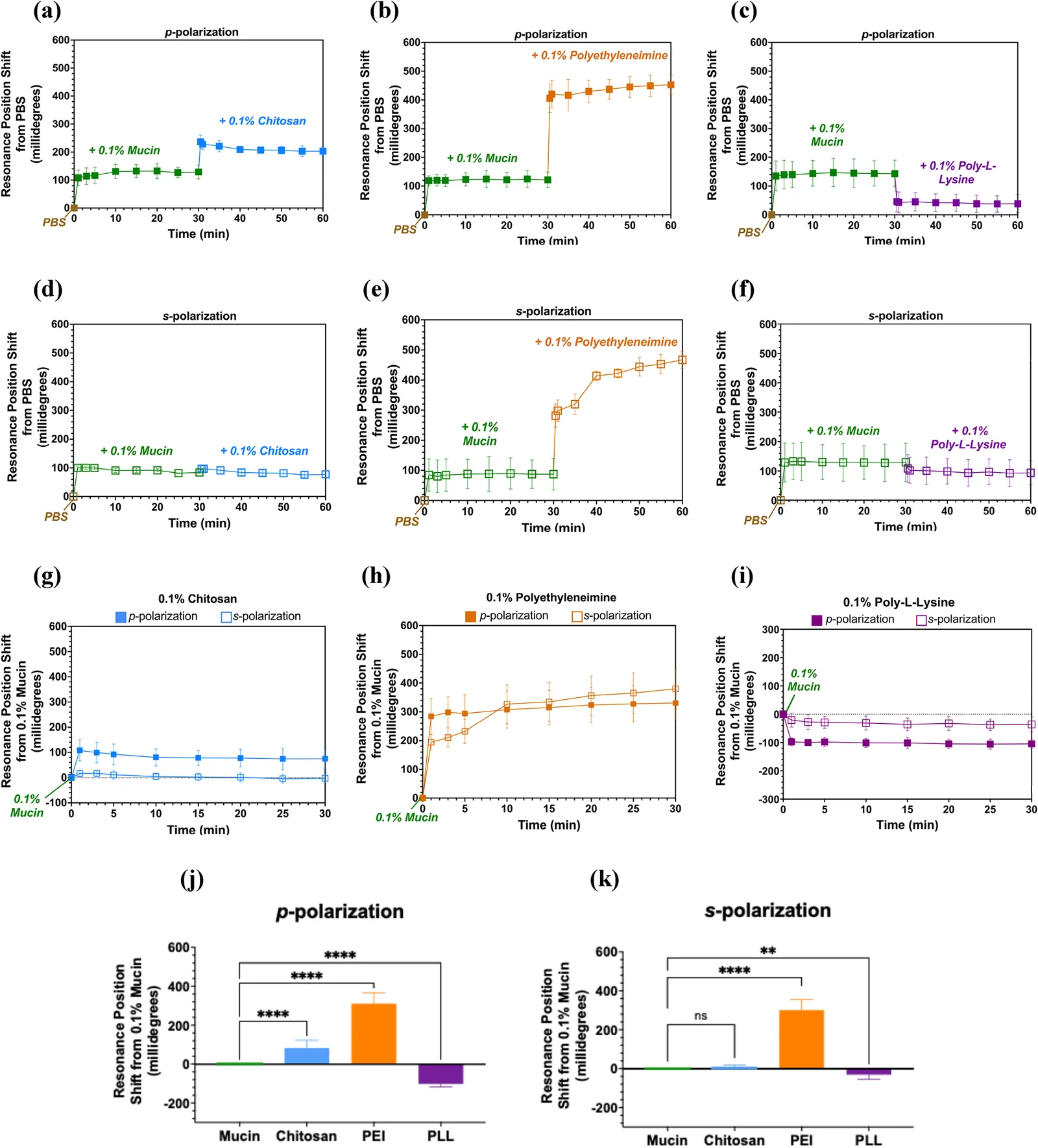
Real-time interaction of mucin and polymers in PWR spectroscopy. (a–c) Resonance position shifts of 0.1% Chitosan, 0.1% w/v PEI, and 0.1% PLL from baseline (PBS) in p-polarization mode, also representing the shift of 0.1% Mucin from PBS baseline. (d–f) Resonance position shifts of 0.1% Chitosan, 0.1% w/v PEI, and 0.1% PLL from baseline (PBS) in s-polarization mode, also representing the shift of 0.1% Mucin from PBS baseline. (g–i) Resonance position shifts of 0.1% w/v Chitosan, 0.1% v/v PEI, and 0.1% v/v PLL from 0.1% w/v Mucin in p- and s-polarization modes combined. (j, k**)** Statistical analysis was performed using one-way ANOVA with Tukey’s test at a 95% confidence interval to determine the significance of PWR angle changes induced by polymer addition to mucin. Statistical significance is represented as follows: *p* < 0.05 (*), *p* < 0.01 (**), *p* < 0.001­(***), *p* < 0.0001­(****); ns, nonsignificant. The mucin-PEI shift was significant in both p- and s-polarization modes (*p* < 0.0001). The mucin–PLL shift was significant in p-polarization (*p* = 0.0085) but not significant in s-polarization (*p* = 0.6631). The mucin-chitosan shift was significant in p- polarization (*p* = 0.0006) but insignificant in s-polarization (*p* > 0.9999). The error bars denote standard deviation, with *n* = 3.

The real-time interaction data for mucin-polymer interactions (*n* = 3) corroborated the observations described above (Figure 5). Chitosan–mucin interaction produced relatively small shifts from the mucin baseline with a mean *p*-shift of 82.30 ± 40.40 millidegrees (CV% = 49.40, *p* < 0.0001) (Figure 5g,5j), and a minimal or negligible *s*-shift of 11.20 ± 7.40 millidegrees (*p* = 0.8823) (Figure 5g,5k). The RPS for PEI stood out when normalized to both PBS (Figure 5b,5e) and mucin (Figure 5h) baselines. The PEI-mucin interaction exhibited the highest RPS among the three polymers, with *p*- and *s*-shifts of 310.3 ± 56.80 millidegrees (CV% = 6.51, *p* < 0.0001) (Figure 5h,5j) and 300.20 ± 54.80 millidegrees (CV% = 25.12, *p* < 0.0001) (Figure 5h,5k), respectively, when normalized to mucin. Contrastingly, PLL interaction with mucin showed an overall decreasing trend when normalized to PBS (Figure 5c,f) and mucin (Figure 5i), with negative RPS values of −101.60 ± 15.60 (CV% = 3.98, *p* < 0.0001) in *p*-polarization (Figure 5i,5j) and *s*-polarization shift of −31.10 ± 23.80 millidegrees (CV% = 51.59, *p* = 0.2556) from the mucin baseline (Figure 5i–5k).

### 3.6 Distinguishing Adsorption and Conformational Changes Using *p*- and *s*-Polarization Modes

The incorporation of *p*- and *s*-polarization modes in PWR spectroscopy provides information related to mass adsorption, as well as the degree of optical anisotropy or ordering in the film upon molecular interactions. To distinguish the effects arising from mass and conformational changes, the graphical analysis method, originally described by Salamon and Tollin (Biophys J. 2004), was applied here.^30^ Using this method, initially, the instrument sensitivity factor (*Sf* = Δ*s*/Δ*p*) was defined, which represents the relative response ratio of *s*-polarization to *p*-polarization for a purely isotropic mass change.^31^ Experimentally, the PWR system used in this study exhibited an *Sf* of 0.33 ± 0.02 (*n* = 9), determined by calculating the PWR shift transition from a bare resonance surface (air scan) to PBS (buffer baseline). This means that an isotropic mass change produces an *s*-shift (Δ*s*) of approximately 33% of the *p*-shift (Δ*p*). *Sf* was then used to determine the slopes for the two orthogonal axes, denoting mass (quadrants I and III) and structural (quadrants II and IV) contributions. The *p*- (Δ*p*) and *s*- (Δ*s*) RPS values from the real-time interaction study (Figure 6) were projected onto the mass (Δ*p_m_*, Δ*s_m_*) and structural (Δ*p*_str_, Δ*s*_str_) axes in a Δ*s* vs Δ*p* plot to yield the magnitudes Δ*m* ( $\sqrt{({\Delta s_{m})}^{2}+\left({\Delta p_{m})}^{2}\right)}$ ) and Δ*str* ( $\sqrt{({\Delta s_{\mathrm{str}})}^{2}+\left({\Delta p_{\mathrm{str}})}^{2}\right)}$ ) values for each condition.^30^ The resulting values gave information about whether an observed effect was mass- or structure-driven (Table 1). The relative mass and structural contributions were calculated by dividing Δ*m* or Δstr by their sum (Δ*m* + Δstr) and expressing each contribution as a percent of the total PWR response.

**Figure 6:**
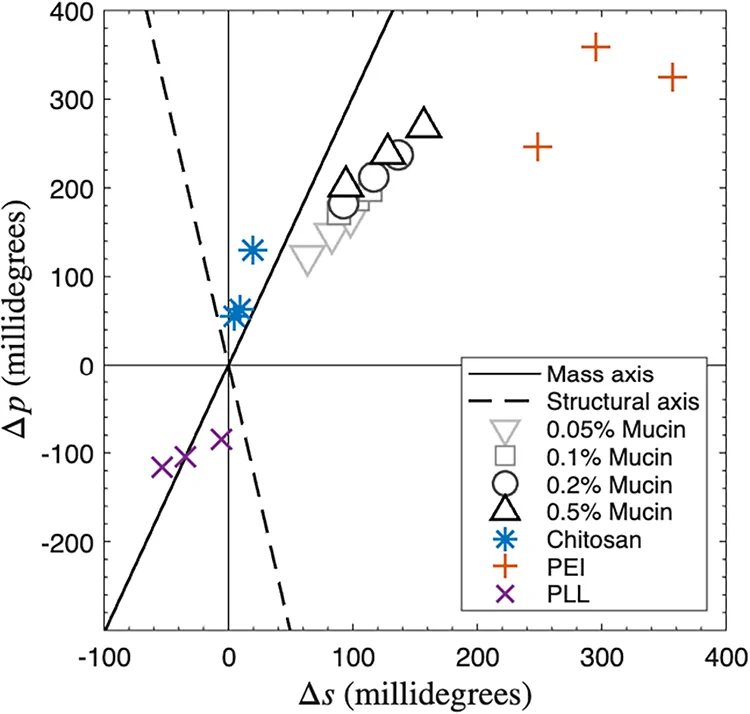
PWR macromolecule interaction analysis of cationic polymers on anionic mucin. The figure shows changes in Δ*p* as a function of Δ*s* (millidegrees) for mucin solutions of increasing concentration (0.05–0.5%) and for polymer (chitosan, PEI, and PLL) interactions with mucin. Solid and dashed lines represent the mass and structural axis, respectively. Symbols correspond to different mucin concentrations (0.05–0.5%) and polymer (chitosan, PEI, and PLL) interactions with mucin. Δ*p* and Δ*s* values for cationic polymers were calculated as the average resonance shifts obtained from the time-course plots. The position of each data point relative to the mass and structural axes denotes the relative contributions of mass accumulation and structural reorganization at the sensor surface. By projecting the data onto the mass and structural axes in the Δ*p* and Δ*s* plane following the graphical analysis approach established by Salamon and Tollin (Biophys J), mass-related (Δ*p*_m_, Δ*s*_m_) and structure-related (Δ*p*_str_, Δ*s*_str_) parameters were obtained (Refer to Table 1). These data were used to determine the magnitude of mass (Δ*m*) and structural (Δstr) contributions for each condition.

**Table 1:** Graphical Analysis of *p*- (Δ*p*) and *s*- (Δ*s*) Polarization Shifts of Mucin (0.05 to 0.5% w/v) and Mucin-Polymer (Chitosan, PEI, and PLL) Interactions

test condition	Δ*p*	Δ*s*	Δ*p*_*m*_	Δ*s*_*m*_	Δ*m*	Δ*p*_str_	Δ*s*_str_	Δstr	Δ*m*%	Δstr%
PBS-water	112.0 ± 6.6	31.0 ± 9.1	99.9 ± 20.7	32.3 ± 6.9	105.2 ± 21.7	12.1 ± 14.8	–2.0 ± 2.4	14.0 ± 12.6	88	12
0.05% Muc-PBS	146.0 ± 21.7	81.6 ± 17.3	213.9 ± 42.5	70.5 ± 14.0	224.8 ± 44.6	–67.5 ± 20.6	11.1 ± 3.4	68.8 ± 20.8	77	23
0.1% Muc-PBS	185.5 ± 13.5	102.2 ± 12.6	268.2 ± 29.8	88.5 ± 9.8	282.5 ± 31.5	–82.7 ± 16.2	13.6 ± 2.7	83.8 ± 16.4	77	23
0.2% Muc-PBS	210.7 ± 28.1	115.1 ± 22.3	302.8 ± 54.3	99.9 ± 17.9	319.2 ± 57.1	–92.8 ± 26.1	15.3 ± 4.3	94.7 ± 26.5	77	23
0.5% Muc-PBS	236.4 ± 33.6	126.4 ± 31.0	334.1 ± 74.2	110.3 ± 24.5	351.9 ± 77.7	–97.7 ± 40.6	16.1 ± 6.7	99.6 ± 41.4	78	22
0.1% mucin-chitosan	82.3 ± 40.4	11.2 ± 7.4	50.2 ± 29.1	16.6 ± 9.6	52.8 ± 30.5	32.2 ± 12.9	–5.3 ± 2.1	32.9 ± 13.1	62	38
0.1% mucin-PEI	310.3 ± 56.8	300.2 ± 54.8	709.8 ± 122.8	234.2 ± 40.5	747.4 ± 129.2	–399.5 ± 83.7	65.9 ± 13.8	405.1 ± 84.8	65	35
0.1% mucin–PLL	–101.6 ± 15.6	–31.1 ± 23.8	–96.8 ± 53.2	–31.9 ± 17.5	101.9 ± 56.3	–14.8 ± 39.1	2.5 ± 6.4	16.4 ± 39.8	86	14

A sensitivity factor (*Sf* = Δ*s*/Δ*p*) of 0.33 ± 0.02 was used for the above analysis. Each measured response vector (Δ*s*, Δ*p*), the resonance shifts in *p*- and *s*-polarization obtained over the time-course, was averaged for cationic polymers and resolved into mass components (Δ*s_m_*, Δ*p_m_*) along the mass axis and structural components (Δ*s*_str_, Δ*p*_str_) along the structural axis in a Δ*s* vs Δ*p* plot to obtain numerical Δ*m* and Δstr values for each test condition. All reported values are in millidegrees, except Δ*m*% and Δstr%. End point resonance shifts were also analyzed using the same graphical analysis framework, yielding similar patterns (Supporting Information Table S.2).

#### 3.6.1 Baseline Response

The response for the PBS shift from water was largely mass-dominated, with Δ*m* responsible for approximately 88% of the total PWR response and a relatively small structural contribution (∼12% of the total response). This result was consistent with minimal interfacial reorganization under buffer exchange conditions and was considered as a baseline response for subsequent comparisons.

#### 3.6.2 Mucin Adsorption

Across the range of mucin concentrations tested (0.05% to 0.5% w/v), the PWR response produced large positive shifts in both *p*- (Δ*p*) and *s*- (Δ*s*) polarization modes, with data points placed in the first quadrant of the Δ*s* vs Δ*p* plot, along the mass axis. Vector decomposition of these points showed that 77–78% of mucin adsorption was dominated by net mass accumulation within the evanescent field, with a secondary contribution from structural changes (∼22–23% of the total response) in the interfacial layer (Table 1).

At 0.05% mucin, Δ*p* and Δ*s* values of 146.0 ± 21.7 and 81.6 ± 17.3 millidegrees, respectively, gave a mass-axis contribution (Δ*m*) of 224.8 ± 44.6 millidegrees (Δ*p*_*m*_ and Δ*s*_*m*_ = 213.9 ± 42.5 and 70.5 ± 14.0 millidegrees, respectively) and a smaller structural component (Δstr) of 68.8 ± 20.8 millidegrees (Δ*p*_str_ and Δ*s*_str_ = −67.5 ± 20.6 and 11.1 ± 3.4 millidegrees, respectively), corresponding to 77% and 23% of the total PWR response, respectively. Similar trends were observed at higher mucin concentrations, with the mass and structural components increasing from Δ*m* = 224.8 ± 44.6 to 351.9 ± 77.7 millidegrees and Δstr = 68.8 ± 20.8 to 99.6 ± 41.4 millidegrees as the mucin concentration increased from 0.05% to 0.5% w/v.

An interesting observation was that the Δ*m*/Δstr ratio remained consistently within the same range (∼3.3–3.8) across all the mucin concentrations tested, indicating that an increase in mucin concentration led to increased mass accumulation at the sensor surface while still maintaining a balance between the mass-driven and structure-driven contributions. Although Δstr (∼22–23% of the total PWR response) represents a smaller fraction of the total PWR response, the presence of the structural component indicates measurable changes in film organization or hydration in the system, rather than pure isotropic mass addition. Δstr did not increase out of proportion at higher mucin concentrations, suggesting that the adsorbed mucin layer reaches a near-stable organizational state as the concentration increases. Based on these findings, mucin adsorption onto the sensor surface likely occurs in two phases: initial rapid surface binding, followed by densification of the mucin layer without major conformational changes. This behavior might be similar to the physiological properties of mucin at mucosal surfaces, where it remains hydrated and structurally organized to form a protective barrier while interacting with other molecules.

#### 3.6.3 Effect of Cationic Polymers on a Mucin-Immobilized Surface

The introduction of cationic polymers to the immobilized mucin layer (0.1% w/v) produced distinct mass and structural responses, showing that each polymer interacts differently with mucin.

Addition of chitosan to the mucin layer resulted in relatively small RPS values (Δ*p* = 82.3 ± 40.40 millidegrees, Δ*s =* 11.20 ± 7.40 millidegrees). Graphical analysis of these shifts produced a comparatively small mass contribution (Δ*m*) of 52.8 ± 30.5 millidegrees; (Δ*p*_*m*_ and Δ*s*_*m*_ = 50.2 ± 29.1 and 16.6 ± 9.6 millidegrees, respectively) and a moderate structural contribution (Δstr) of 32.9 ± 13.1 millidegrees (Δ*p*_str_ and Δ*s*_str_ = 32.2 ± 12.9 and – 5.3 ± 2.1 millidegrees, respectively), compared to the other polymers tested. The Δstr was approximately 38% of the total response, indicating that chitosan interaction with mucin is associated with interfacial structural changes rather than pure mass addition. The direction and magnitude of Δstr point toward decreased anisotropy. The projected structural components (Δ*p*_str_ and Δ*s*_str_) were oriented in the opposite direction compared to mucin alone adsorption (Δ*p*_str_ and Δ*s*_str_ = −82.7 ± 16.2 and 13.6 ± 2.7, respectively), suggesting a decrease in optical anisotropy, as a result of structural reorganization of the mucin layer. This response is consistent with the known mucoadhesive nature of chitosan, which reorganizes the mucin layer, increasing residence time.^32^

PEI-mucin interaction showed a significantly different interaction profile with large positive shifts in both polarizations (Δ*p* = 310.30 ± 56.8 millidegrees, Δ*s* = 300.20 ± 54.80 millidegrees). Graphical decomposition showed a dominant mass contribution Δ*m* = 747.4 ± 129.2 millidegrees (Δ*p*_*m*_ and Δ*s*_*m*_ = 709.8 ± 122.8 and 234.2 ± 40.5 millidegrees, respectively), which was substantially higher than the other polymers tested. Although Δ*m* accounted for approximately 65% of the total response, structural contributions (Δstr = 405.1 ± 84.8 millidegrees) were also significant (∼35% of the total response). The projected structural components (Δ*p*_str_ and Δ*s*_str_ = −399.5 ± 83.7 and 65.9 ± 13.1 millidegrees, respectively) were oriented in the same direction compared to mucin alone adsorption (Δ*p*_str_ and Δ*s*_str_ = −82.7 ± 16.2 and 13.6 ± 2.7 millidegrees, respectively). However, the substantially larger magnitude of the structural component compared to mucin indicated a significant structural change of the mucin layer, indicating an increase in optical anisotropy within the mucin network. Overall, the combination of large Δ*m* and Δstr indicates that PEI penetrates strongly into the mucin layer, leading to significant mass accumulation along with increased anisotropy of the mucin layer. Physiologically, this interaction could mean strong polymer binding to the mucus layer, with potential risk of toxicity due to epithelial exposure.^33,34^

PLL produced negative shifts in both *p*- and *s*-polarization modes (Δ*p* = −101.60 ± 15.60 millidegrees, Δ*s* = −31.10 ± 23.80 millidegrees), indicative of net mass loss from the surface. Graphical analysis produced a moderate mass component of Δ*m* = 101.9 ± 56.3 millidegrees and a very low structural component of Δstr = 16.4 ± 39.8 millidegrees, compared to chitosan and PEI. The mass and structural components of mucin-PLL interaction contributed to approximately 86% and 14% of the total response, respectively. The negative mass components (Δ*p*_*m*_ and Δ*s*_*m*_ = −96.8 ± 53.2 and −31.9 ± 17.5 millidegrees, respectively) suggest partial removal of mucin from the surface, while the accompanying structural components (Δ*p*_str_ and Δ*s*_str_ = −14.8 ± 39.1 and 2.5 ± 6.4 millidegrees, respectively) indicate a very minor increase in optical anisotropy within the remaining film. The directionality of the structural shift matches that observed for PEI, suggesting that both polymers induce similar structural changes, although with different magnitudes and opposite effects on mass. These observations support an important biological implication of PLL: while it may enhance drug permeability by partially removing the mucosal barrier, the compromised integrity of the mucosal surface may make PLL cytotoxic to underlying epithelial cells, especially at higher concentrations.^35^

Overall, the dual *p*- and *s*-polarization PWR measurements provide a deeper understanding of mucin-polymer interactions. Graphical analysis of *p*- and *s*-shifts showed unique patterns of interaction of cationic polymers (chitosan, PEI, and PLL) with mucin. Chitosan interaction with mucin led to a decrease in anisotropy in addition to mass adsorption, though the magnitude of these effects was low. The mass-dominant interaction of PEI with mucin was accompanied by increased anisotropy, indicative of substantial structural change of the mucin layer. PLL caused net mass loss with limited structural change, potentially due to partial displacement or thinning of the mucin layer.

Biologically, these results provide three different mucus outcomes: mucoadhesive restructuring (chitosan), strong polymer accumulation with perturbation of the mucin layer (PEI), and barrier weakening and displacement through partial mucin removal (PLL), each of which has a direct effect on retention and penetration in mucosal drug delivery. This approach helped distinguish polymers that simply adsorb on the mucosal surface from those that reorganize or destabilize the mucin layer, thereby providing a much deeper picture of how these polymers interact with the mucosal barrier.

### 3.7 Physicochemical Characterization

The pH, hydrodynamic diameter, polydispersity index (PDI), and ζ-potential (ZP) of the test solutions (0.1% w/v mucin, 0.1% w/v chitosan, 0.1% v/v PEI, and 0.1% v/v PLL) were measured to assess whether these physiochemical properties influenced the nature of the mucin-polymer interactions described above (Section 3.5). As expected, the average pH of mucin (7.22 ± 0.01) was comparable to that of PBS (7.41 ± 0.03). Chitosan, due to its acidic nature, gave a lower pH value of 4.21 ± 0.03. PEI, on the other hand, had a highly basic pH of 11.01 ± 0.01. The pH of PLL was 4.85 ± 0.02 (Figure 7a). The average hydrodynamic diameter of mucin was found to be around 473.7 ± 43.00 nm. Chitosan had a hydrodynamic diameter of 269.8 ± 5.50 nm. Contrastingly, PEI had the smallest hydrodynamic diameter at 10.25 ± 0.20 nm due to its highly compact structure, whereas PLL had an intermediate hydrodynamic diameter of 87.50 ± 30.00 nm (Figure 7c). Within the test solutions, PEI showed the lowest PDI (0.25 ± 0.03), signifying a homogeneous distribution of particle size, whereas the PDI of chitosan (0.73 ± 0.08) and mucin (0.45 ± 0.22) was more than twice that of PEI, suggesting more polydisperse solutions. PLL had the highest PDI (1.0 ± 0.01) among the test solutions, reflecting greater aggregation (Figure 7d). The ZP studies indicated that mucin had a slightly negative ζ-potential (−0.96 ± 1.35 mV). PEI had the lowest positive ZP at +0.84 ± 0.35 mV, whereas PLL showed the highest positive ZP at +29.57 ± 3.98 mV. Chitosan had a moderate positive ZP of +9.20 ± 1.14 mV (Figure 7b). All measurements of pH, hydrodynamic diameter, PDI, and ZP were conducted in triplicate (*n* = 3).

**Figure 7:**
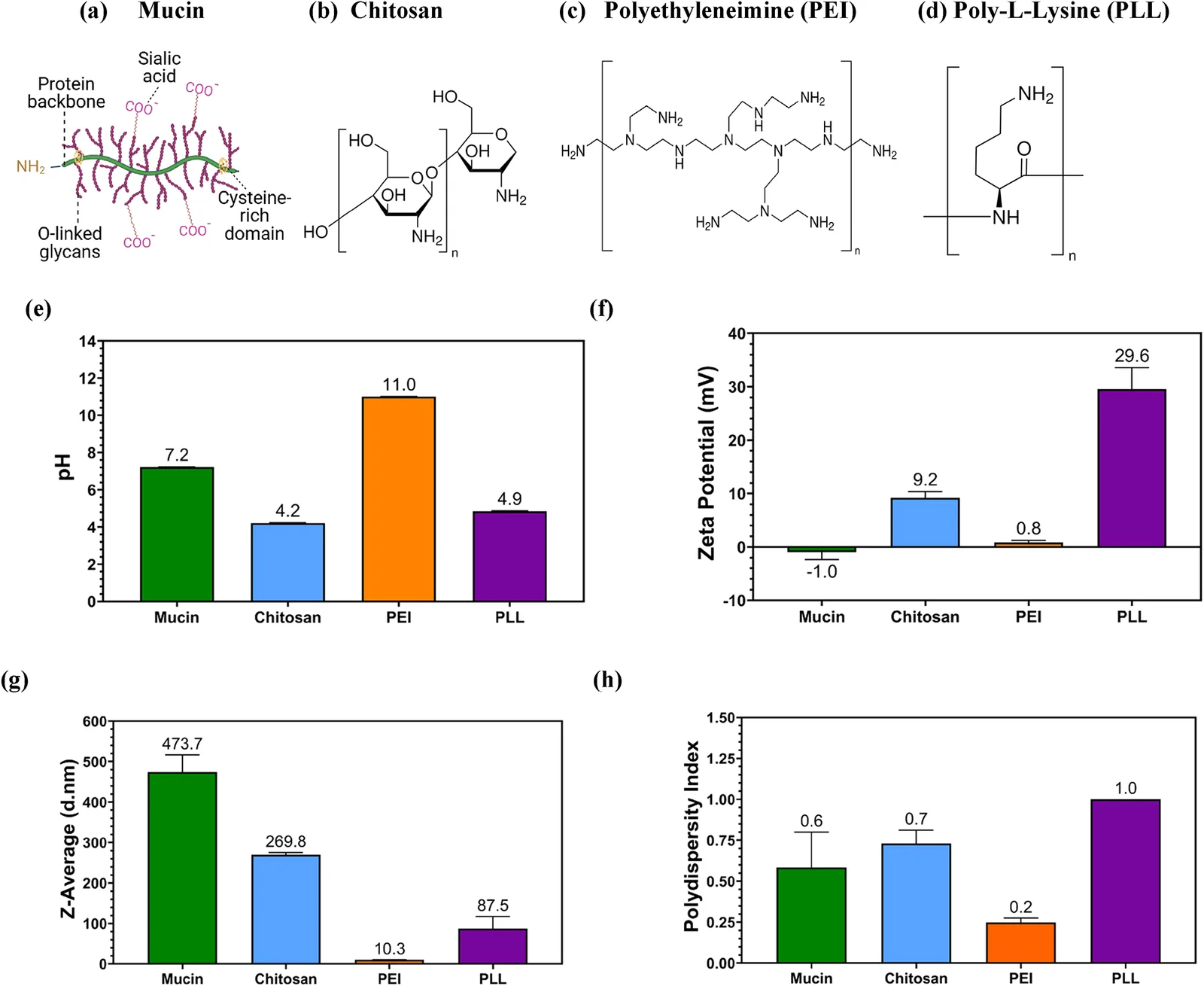
(a–d) Representative chemical structures of (a) mucin, (b) chitosan, (c) PEI, and (d) PLL. (e–h) Physicochemical characterization of mucin and polymer solutions, including (e) pH, (f) ζ-potential, (g) hydrodynamic diameter, and (h) polydispersity index (PDI). The error bars denote standard deviation, with *n* = 3.

Although physicochemical characterization of the test solutions revealed no definitive correlation with the mucin-polymer interaction behavior observed by PWR spectroscopy, certain trends emerged. The strongly mass-dominated mucin interaction shown by branched PEI may be partly attributed to its small hydrodynamic diameter (∼10 nm), which can facilitate close packing with the mucin network and enable multivalent electrostatic interactions with mucin chains.^36^ On the other hand, the comparison of chitosan (∼270 nm) and PLL (∼87.45 nm) highlights that particle size alone is not predictive of interaction behavior. PLL, though having a much smaller size than chitosan, produced net negative shifts relative to the mucin baseline, indicating a loss of interfacial mass consistent with partial removal or thinning of the mucin layer. Chitosan, on the other hand, produced smaller positive shifts from mucin and showed mass accumulation and structural reorganization of the mucin layer. Additionally, chitosan and PLL, despite having similar pH values (∼4.2 and ∼4.9, respectively), showed divergent trends when interacting with mucin, indicating that polymer structure (branched vs linear), reactive groups, charge distribution, etc., may play significant roles in mucin-polymer binding, when compared to bulk properties like pH and ζ-potential.

### 3.8 Mechanistic Interpretation of Mucin Binding Behavior with Cationic Polymers

Mucins are large, polyanionic compounds with peptide backbones that are densely decorated with O-linked glycans, which terminate in sialic acid and cysteine-rich domains.^4,36^ Due to the presence of these terminal anionic residues, at near-neutral pH, mucin attains a net negative charge.^37^ For the same reason, electrostatic repulsive forces come into action and cause the mucin chains to adopt a random coil-like conformation in solution.^38,39^ When cationic polymers were introduced to the negatively charged mucin layer, it was expected that the polymers would adsorb to the mucin layer to some extent due to electrostatic forces.

Chitosan, a linear polysaccharide, when introduced onto the mucin layer, resulted in a moderate but significant increase in the resonance position by 82.30 ± 40.40 millidegrees (*p* < 0.0001) relative to the immobilized mucin in *p*-polarization mode and no significant change in *s*-polarization mode (11.20 ± 7.40 millidegrees (*p* = 0.8823)) (Figure 5j,5k). The presence of primary amines in its glucosamine units provides chitosan (p*K*_a_ ≈ 6.2) with a positive charge under slightly acidic conditions. These amine groups on the linear chitosan undergo protonation, which leads to expansion of its chains.^40^ This, in turn, provides the opportunity for its −NH_3_^+^ groups to form electrostatic interactions with the −COO^–^ and −SO_3_^–^ groups present in mucin, available from sialic acid and sulfated glycan residues of mucin, respectively.^41^ This behavior is attributed to electrostatic interactions and hydrogen bonding between mucin and chitosan, along with the removal of repulsive electrostatic forces between the two polymers.^42–46^ Thus, in addition to mucin adsorption, chitosan may also lead to a change in mucin rheology as it releases water from the mucin film, causing the mucin film to collapse partially to satisfy the charges.^46^ Altogether, these established electrostatic and hydrogen-bonding interactions provide a mechanistic basis for the response observed in the current study, where graphical analysis shows that the chitosan–mucin interaction generates structural vectors (Δ*p*_str_ and Δ*s*_str_) opposite in direction to mucin adsorption alone, suggesting a decrease in optical anisotropy and structural reorganization of the mucin film.

Contrastingly, introduction of the cationic branched polyethylenimine (PEI) resulted in a significantly higher RPS with median shifts of 310.30 ± 56.80 millidegrees (*p* < 0.0001) and 300.20 ± 54.80 millidegrees (*p* < 0.0001) for *p*- and *s*-polarization, respectively, (Figure 5j,5k), indicating extensive adsorption or mixing of the polymer with mucin, compared to chitosan. This is attributed to the high density of primary, secondary, and tertiary amines in the structure of PEI (p*K*_a_ = 8.18 to 9.94), which induces strong affinity and binding to the mucin surfaces.^47–49^ Patil et al. showed that the branched PEI has the ability to contract the randomly coiled structure of mucin by interacting and binding strongly with the mucin molecules, particularly at the terminals, resulting in increased lubrication.^36^ Nikogeorgos et al, suggested that PEI is able to achieve its optimum lubrication synergy with mucin due to its smaller hydrodynamic size and compact structure with no bulky side groups present on its branched chains.^49^ In the context of this study, the large Δ*m* (∼65% of the total PWR response) and Δstr (∼35% of the total response) of PEI, obtained through graphical analysis, indicate strong mucin penetration, mass accumulation, and increased anisotropy of the mucin film. This is likely due to the presence of multiple binding sites (positively charged amine groups) on PEI, which enables it to interact with various sites in the mucin network. This, in turn, may collapse or contract the randomly coiled structure of mucin at the site of the polymer interaction, possibly also neutralizing the net local charge of the mucin layer.

Considerably different from what was observed with the linear chitosan and branched PEI, poly-l-lysine (PLL), a linear cationic polymer (p*K*_a_ ≈ 9),^50^ produced a negative shift (mean RPS of −101.60 ± 15.60 millidegrees (*p* < 0.0001) and −31.10 ± 23.80 millidegrees (*p* = 0.2556) for *p*- and *s*-polarization, respectively) when introduced to immobilized mucin (Figure 5j,5k). The shifts were strongly oriented in the negative direction, away from the mucin curve (Figure 5c,5f), indicating a loss of mass or desorption. This finding was unexpected because the positively charged PLL would generally be expected to interact strongly with the negatively charged mucin. The Δ*m* and Δstr (∼86% and ∼14% of the total PWR response, respectively) suggest that PLL may disrupt the mucin layer in a way that reduces the refractive index or optical thickness at the interface. One possible explanation for this phenomenon is that the linear, flexible backbone of PLL allows its penetration into the mucin layer, which could lead to the removal of loosely bound mucin or partial removal of the immobilized layer.^50^ Because the PWR angle depends on both the refractive index and sample thickness, a decrease in mass would shorten the optical path length and result in negative shifts, as observed with PLL-mucin interaction in the present study.

Here, we observed three different variations of mucin-polymer interactions, potentially driven primarily by an electrostatic force. Unlike the linear polymers chitosan and PLL, branched PEI produced a substantial increase in RPS from mucin, likely due to its small size and the high density of positively charged amines (primary, secondary, and tertiary) available to interact with the negatively charged mucin.^43,44^ On the other hand, chitosan and PLL displayed noticeably different interaction profiles with mucin. Chitosan, with its rigid cyclic backbone and restricted chain flexibility, produced a smaller overall RPS than PEI, consistent with comparatively lower mass accumulation at the mucin interface, while still producing a notable structural contribution indicative of mucin layer reorganization. Contrastingly, PLL, with its flexible linear structure and extended aliphatic side chains, appeared to induce partial desorption or thinning of the mucin layer, as reflected by the highly negative RPS relative to the mucin baseline. These findings underscore that not all cationic polymers interact with mucin in a similar manner. Chitosan primarily induces reorganization of the mucin layer with decreased optical anisotropy; PLL leads to partial mucin removal; and PEI penetrates the mucin layer, resulting in substantial mass accumulation and structural perturbation.

### 3.9 Potential Applications and Future Directions

Future applications of this PWR technique could leverage immobilized mucin on the PWR sensor surface as a platform to quantitatively assess polymer-mucin interactions. This approach has the potential to streamline the optimization of drug formulations by connecting the degree of mucin binding or structural reorganization to functional outcomes such as adhesion, retention, and permeability. Future studies will further explore how variations in polymer concentration influence these interactions and the resulting changes in the mucin network properties. Eventually, this approach could be modified to assess a wide range of biological interfaces to understand how components in a formulation modify surface structure and permeability and, thereby, to guide the design of more effective drug delivery systems to enhance therapeutic delivery outcomes.

## 4 Conclusions

PWR is a surface-sensitive technology that monitors molecular-level adsorption and structural organization. The key advantage of this technique lies in its ability to acquire both *p*- and *s*-polarization data, which provides unique insight into the anisotropy of biological events at the surface. In this study, we used PWR spectroscopy with its dual polarization modes to capture adsorption (*p*-polarization) and structural changes (*s*-polarization) of anionic mucin (from porcine stomach), a complex glycoprotein abundant in mucosal linings, during its interaction with three cationic polymers commonly used in drug delivery: polyethylenimine (PEI), poly-l-lysine (PLL), and chitosan.

Using mucin as a model surface layer, PWR spectroscopy captured polarization-dependent behaviors that provide insights about the mucin layer density, organization, and hydration. Increasing mucin concentration led to a concentration-dependent increase in *p*-polarization shifts, consistent with the increase in optical density of the film, while the comparatively smaller *s*-polarization shift denoted limited structural changes of the mucin layer once it reached equilibrium. Introduction of the cationic polymers onto the immobilized mucin surface caused unique PWR angle shifts from mucin. The *p*- and *s*-polarization measurements distinguished the differences in the nature of mucin-polymer interaction, the magnitude of which likely depended on the architecture of the polymer, such as branched versus linear chains, molecular size, reactive groups, etc.

Chitosan, PEI, and PLL interactions with mucin produced moderate, large positive, and negative shifts, respectively, in both polarization modes. Graphical analysis of the *p*- and *s*-shifts revealed unique effects of mucin-polymer interactions arising from differences in mass accumulation and structural changes. Among the polymers tested, PEI produced the largest magnitudes of both mass and structural contributions and exhibited a mass-dominated response, with the structural component indicating an increase in anisotropy. Chitosan, on the other hand, showed comparatively smaller Δ*m* and Δstr values, with the structural vector oriented opposite to mucin, suggesting a polymer-induced decrease in anisotropy. PLL produced a negative mass component with a relatively small structural component, indicating partial removal or thinning of the mucin layer.

Overall, these results show that the combined *p*- and *s*-polarization PWR measurements help differentiate between polymers that simply adsorb on the surface and those that reorganize or destabilize the mucin barrier. The mucin-coated PWR sensor platform described herein offers a sensitive approach to evaluate and differentiate mucin-polymer interactions. This technology can be readily extended to excipients and drug delivery platforms to understand their interactions with mucin and guide the development of optimized formulations within shorter experimental timelines.

## Supplementary Material



## Data Availability

The data obtained from these studies are presented within the manuscript.

## Notes

pH stability of mucin under different storage conditions; graphical analysis of end point PWR polarization shifts for mucin-polymer interactions (PDF)
