# Supplementary material for: Label-Free Plasmon-Waveguide Resonance Analysis of Mucin Network Remodeling by Cationic Polymers
Source: ACS Meas Sci Au. 2026 Apr 9;6(4):983–97. doi: 10.1021/acsmeasuresciau.6c00012 (PMC13495493; doi:10.1021/acsmeasuresciau.6c00012)
Supplement: Supplementary file 1 [file tg6c00012_si_001.pdf]

Supporting information

Label-Free Plasmon-Waveguide Resonance Analysis of Mucin Network Remodeling by Cationic Polymers

Shilpa E. George[1,3], Amanda M. Ratajczak[2], and Uday B. Kompella [1,3,4,5*]

[1]*Department of Pharmaceutical Sciences, University of Colorado Anschutz Medical Campus, Aurora, CO 80045, United States*

[2] *Mainline Scientific LLC, Malvern, PA 19355, United States*

[3] *Department of Ophthalmology, University of Colorado Anschutz Medical Campus, Aurora, CO 80045, United States*

[4] *Department of Bioengineering, University of Colorado Anschutz Medical Campus, Aurora, CO 80045, United States*

[5]*Colorado Center for Nanomedicine and Nanosafety, University of Colorado Anschutz Medical Campus, Aurora, CO, USA 80045, United States*

*Corresponding Author:

Uday B. Kompella, PhD.,
Department of Pharmaceutical Sciences
University of Colorado Anschutz Medical Campus
12850 East Montview Boulevard, C238-V20
Aurora, CO 80045
(Email: uday.kompella@cuanschutz.edu)

Table S.1. Stability of mucin preparations assessed by pH over 8 days at room temperature and 4 °C.

pH at Room Temperature (RT)

Time (h)	PBS	0.05% Mucin	0.1% Mucin	1% Mucin	2% Mucin
0	7.41	7.41	7.39	7.37	7.34
2	7.36	7.38	7.36	7.35	7.33
4	7.36	7.35	7.34	7.29	7.26
24	7.23	7.21	7.18	7.13	7.11
48	7.23	7.22	7.20	7.18	7.17
72	7.22	7.21	7.19	7.15	7.14
120	7.25	7.23	7.18	6.87	6.77
144	7.25	7.20	7.18	6.85	6.79
168	7.23	7.17	7.18	6.84	6.81
172	7.23	7.23	7.22	6.98	6.95
196	7.23	7.22	7.22	6.88	6.90
SD	0.07	0.08	0.08	0.21	0.22
CV (%)	0.94	1.12	1.12	2.90	3.07

pH at 4°C

Time (h)	PBS	0.05% Mucin	0.1% Mucin	1% Mucin	2% Mucin
0	–	–	–	–	–
2	7.15	7.36	7.35	7.34	7.30
4	7.32	7.30	7.27	7.22	7.18
24	7.16	7.15	7.14	7.12	7.08
48	7.22	7.22	7.15	7.17	7.19
72	7.22	7.22	7.16	7.16	7.15
120	7.19	7.19	7.10	7.07	7.05
144	7.10	7.12	7.10	7.05	7.03
168	7.04	7.11	7.09	7.03	7.00
172	7.20	7.20	7.20	7.19	7.19
196	7.21	7.21	7.20	7.17	7.15
SD	0.08	0.08	0.08	0.09	0.09
CV (%)	1.05	1.06	1.15	1.28	1.28

pH values of PBS and mucin solutions (0.05% to 2% w/v) were measured at multiple time points over 196 hours and under two storage conditions: room temperature (top) and refrigerated at 4 °C (bottom). PBS served as the control. Values represent mean pH measurements over time, and standard deviations (SD) are shown in the final row of each table.

Table S.2. *Graphical analysis of the endpoint measurement of p- (Δp) and s- (Δs) polarization shifts of mucin (0.05 to 0.5 % w/v) and mucin-polymer (chitosan, PEI and PLL) interactions using a sensitivity factor* $S_f = \Delta s/\Delta p = 0.33$.

Test Condition	Δ*p*	Δ*s*	Δp_m	Δs_m	Δ*m*	Δp_{str}	Δs_{str}	Δ*str*	Δm %	Δstr %
**PBS-Water**	104.40	21.40	78.03	25.75	82.17	26.37	-4.35	26.72	75.46	24.54
	116.10	39.60	118.70	39.17	125.00	-2.60	0.43	2.64	97.93	2.07
	115.50	31.97	103.09	34.02	108.55	12.41	-2.05	12.58	89.61	10.39
**0.05% Muc-PBS**	123.34	63.51	169.42	55.91	178.40	-46.08	7.60	46.70	79.25	20.75
	148.00	83.10	217.21	71.68	228.73	-69.21	11.42	70.15	76.53	23.47
	166.76	98.19	253.95	83.80	267.42	-87.19	14.39	88.37	75.16	24.84
**0.1% Muc-PBS**	171.04	88.94	236.69	78.11	249.24	-65.65	10.83	66.54	78.93	21.07
	187.50	103.66	271.91	89.73	286.34	-84.41	13.93	85.56	76.99	23.01
	198.06	113.88	296.08	97.71	311.79	-98.02	16.17	99.35	75.84	24.16
**0.2% Muc-PBS**	181.74	92.36	247.17	81.56	260.28	-65.43	10.80	66.31	79.70	20.30
	212.65	116.62	306.48	101.14	322.74	-93.83	15.48	95.10	77.24	22.76
	237.66	136.38	354.74	117.06	373.55	-117.08	19.32	118.66	75.89	24.11
**0.5% Muc-PBS**	202.00	94.70	258.65	85.35	272.37	-56.65	9.35	57.41	82.59	17.41
	238.40	127.88	337.81	111.48	355.73	-99.41	16.40	100.75	77.93	22.07
	268.90	156.56	405.92	133.95	427.45	-137.02	22.61	138.87	75.48	24.52
**0.1% Mucin-Chitosan**	121.10	18.90	122.13	40.30	128.61	-1.03	-21.40	21.42	85.72	14.28
	45.00	-5.40	40.23	13.28	42.36	4.77	-18.68	19.28	68.72	31.28
	51.80	-2.20	55.90	18.45	58.86	-4.10	-20.65	21.05	73.66	26.34
**0.1% Mucin-PEI**	364.50	372.60	472.41	155.89	497.46	-107.91	216.71	242.05	67.27	32.73
	365.00	453.60	507.04	167.32	533.94	-142.04	286.28	319.60	62.56	37.44
	262.80	313.60	273.05	90.11	287.52	-10.25	223.49	223.72	56.24	43.76
**0.1% Mucin-PLL**	-120.60	-62.10	-136.18	-44.94	143.40	15.58	-17.16	23.18	86.08	13.92
	-103.00	-36.90	-112.07	-36.98	118.01	9.07	0.08	9.07	92.86	7.14
	-90.50	-8.50	-82.96	-27.38	87.37	-7.54	18.88	20.34	81.12	18.88

Each measured response vector (Δs, Δp) was resolved into a mass component (Δs_m, Δp_m) along the mass axis and a structural component (Δs_{str}, Δp_{str}) along the structural axis in a Δs vs Δp plot to obtain numerical Δm and Δstr values for each test condition. All reported values are in millidegrees, except Δm% and Δstr.
